# The Impact of Crowd Noise on Officiating in Muay Thai: Achieving External Validity in an Experimental Setting

**DOI:** 10.3389/fpsyg.2012.00346

**Published:** 2012-09-14

**Authors:** Tony Myers, Nigel Balmer

**Affiliations:** ^1^Newman University CollegeBirmingham, UK; ^2^University College LondonLondon, UK

**Keywords:** home advantage, crowd noise, Muay Thai, external validity, officiating, judging

## Abstract

Numerous factors have been proposed to explain the home advantage in sport. Several authors have suggested that a partisan home crowd enhances home advantage and that this is at least in part a consequence of their influence on officiating. However, while experimental studies examining this phenomenon have high levels of internal validity (since only the “crowd noise” intervention is allowed to vary), they suffer from a lack of external validity, with decision-making in a laboratory setting typically bearing little resemblance to decision-making in live sports settings. Conversely, observational and quasi-experimental studies with high levels of external validity suffer from low levels of internal validity as countless factors besides crowd noise vary. The present study provides a unique opportunity to address these criticisms, by conducting a controlled experiment on the impact of crowd noise on officiating in a live tournament setting. Seventeen qualified judges officiated on thirty Thai boxing bouts in a live international tournament setting featuring “home” and “away” boxers. In each bout, judges were randomized into a “noise” (live sound) or “no crowd noise” (noise-canceling headphones and white noise) condition, resulting in 59 judgments in the “no crowd noise” and 61 in the “crowd noise” condition. The results provide the first experimental evidence of the impact of live crowd noise on officials in sport. A cross-classified statistical model indicated that crowd noise had a statistically significant impact, equating to just over half a point per bout (in the context of five round bouts with the “10-point must” scoring system shared with professional boxing). The practical significance of the findings, their implications for officiating and for the future conduct of crowd noise studies are discussed.

## Introduction

Home advantage is defined as “the consistent finding that home teams in sport competitions win over 50 % of games played under a balanced home and away schedule” (Courneya and Carron, 1992, p.14). The phenomenon is well established in both academic literature and in popular culture across a range of sports and time periods (Pollard and Pollard, 2005). While different sports vary in their susceptibility to the home advantage effect, evidence from a range of sports and eras suggests that the home team or competitor wins around 60% of all sporting contests (Jamieson, 2010). Given its ubiquity, the home advantage phenomenon has received academic attention from a range of different disciplines and been explored using different methodologies ranging from statistical consideration of archive data to experimental investigation of cause and effect.

Several researchers consider crowd influences important in the home advantage phenomena. In their seminal study, Schwartz and Barsky (1977) claimed that the advantage gained from playing at home was the result of the social support offered by partisan fans. Equally, vocalized dissatisfaction has been found to influence performance and ultimately home advantage. When crowds voice their displeasure with a team’s performance by booing, it appears the home team tends to respond by playing better, which in turn leads to an advantage over the away team (Greer, 1983). Greer concluded that during both normal and booing crowd behavior conditions, the home team’s performance was better than that of the visiting team. However, during those instances when the crowd was booing, the home team’s superiority increased further.

Differing methodologies have been used to explore the impact of the crowd on officials. These include the retrospective analysis of archival data, utilization of naturally occurring experiments, and specifically designed experiments. Analyses of archival data offer a number of benefits when exploring crowd effects on home advantage. In particular, large amounts of data spanning numerous years can be used. For example, some of the studies have investigated the results of sports competitions that span as many as 90 years (Balmer et al., 2001). However, equally, there are potential issues when considering its use. The evidence generated from this type of data provides useful supporting evidence to the findings of other specifically designed studies. Nevertheless, solely using this type of data to draw conclusions regarding crowd influences on officials has to be questioned. When examining the evidence generated from the home advantage literature using such data, one has to be mindful of what a researcher feels they should find or “prove” and then assess whether there has been any “fitting” of the data or evidence to suit a particular hypothesis. The choice of variables and the method of analysis can generate different findings from the same data set.

On occasion, unique situations arise where it has been possible to observe the impact of “naturally occurring” crowd conditions. For example, when for particular reasons teams have had to play without spectators present (e.g., Moore and Brylinsky, 1993; Pettersson-Lidbom and Priks, 2007). Pettersson-Lidbom and Priks (2007) used such an opportunity in Italian soccer to compare crowd and no crowd conditions and how this affected officials’ behavior. Italian soccer teams playing at stadia whose safety standards had been declared deficient, had to play their home games temporarily without spectators. This allowed a direct comparison between games with no spectators on the one hand, and games with very large numbers of spectators on the other. The researchers found that referees punished away players more severely and home players more leniently when the games were played in front of a crowd of supporters. The authors suggested that social pressure might have caused officials to consciously alter their own behavior to appease home supporters, by punishing away players more harshly, yet conversely treating home players more leniently.

Such chance occurrences offer an interesting insight into crowd effects in ecologically valid settings. However, given their observational nature they do not allow the specific manipulation of conditions or any control over extraneous variables. So, these studies can at best be considered pre-experimental in terms of their research design with no deliberate manipulation of variables (Creswell, 2009). As such, the influences on officials and the mechanisms put forward to explain the influence are at best speculative. To demonstrate cause and effect, experiments are undoubtedly necessary.

In the case of soccer, experimental studies have demonstrated the significant impact that crowd noise has on influencing refereeing decisions. Nevill et al. (1999, 2002) and Balmer et al. (2007) conducted laboratory-based experiments where participants made refereeing decisions in the presence of crowd noise (noise condition), or without crowd noise (a no noise condition). In all experiments, the presence of crowd noise resulted in an imbalance of decisions in favor of the home side when compared to the “no noise” condition. Nevertheless, these studies suffer from a number of methodological shortcomings.

One contentious issue highlighted by other researchers investigating crowd influences on officials (Sutter and Kocher, 2004; Unkelbach and Memmert, 2010) is that Nevill, Balmer, and Williams study only used incidents from a single game. The video footage of 47 challenges presented to 40 referees were taken from a single English Premier League soccer match (Liverpool versus Leicester City). The criticism leveled at this approach centered on it being impossible to determine whether the effect found represented merely a team advantage, or that of a more general home advantage.

Unkelbach and Memmert (2010) addressed this specific methodological issue through their own crowd noise experiment. They showed video footage from 56 different soccer games together with recorded crowd reactions to fouls at different stadiums. Rather than differentiating between, “crowd noise” and “no crowd noise,” a duality they considered unrealistic, they opted instead to accompany the footage of each challenge with utilizing both “high” and “low” volumes of crowd noise. The use of different scenes of challenges from different teams mean they were able to eliminate any possible team affect.

Unkelbach and Memmert (2010) support the notion that a different mechanism exists in comparison to the motivational hypothesis postulated by the previous research team. This is despite their research being complimentary to that of Nevill and his colleagues. Although they do acknowledge a possible motivational influence toward the home bias in general, their results could be regarded as pointing more strongly toward referees using crowd noise as a cue in their decision-making by correlating foul severity with crowd noise. Whatever the specific nature of influence, the experimental noise studies appear to clearly demonstrate the influence of crowd noise on referee decisions within a laboratory setting. Whether or not such evidence offers clear cut support for a crowd noise effect in live sports settings, is contentious and one open to a debate.

Sports officials who judge incidents on a video screen accompanied by recorded crowd noise, are clearly not in the same situation as they would be when officiating at a live sports event in front of a real crowd. Unkelbach and Memmert (2010) acknowledge this issue with external validity. In contrast, Nevill et al. (2002) claim strong external validity in their experiment. While the claim has some merit given the decisions of participants in the noise condition mirrored those of match referees, the use of recorded crowd noise and video footage has seriously hampered the external validity of the findings in this area.

The experimental methodology applied in studies means that crowd noise effects are attributable to the noise intervention (high internal validity). Nonetheless, officiating in laboratory settings of this type severely limits the external validity of the studies. First, officiating before a screen with recorded crowd noise differs markedly from that occurring in live matches or boxing bouts. A sports official’s experience therefore is different in a laboratory setting where recorded sound played through headphones differs both in volume and character to live noise. Second, in the laboratory setting, none of the decisions made have direct implications for teams or players. Conversely, in actual live events, decisions have a real bearing on the outcomes of matches. For example, in the present study the decisions made by participants directly decided the outcome of matches. Although these studies (Nevill et al., 1999, 2002; Balmer et al., 2007; Unkelbach and Memmert, 2010) successfully, demonstrate that crowd noise has an impact on the laboratory-based decisions of study participants, it is unclear as to the extent to which these findings can be generalized to actual live sports settings.

The general approach used in the present study is comparable to that used by Nevill et al. (1999, 2002). Participants make judgments either in the presence of, or absence of partisan home support, with the study aiming to assess the change in judgments (presumably in favor of the home competitor) attributable to crowd noise. In the current study, we examined the impact of crowd noise on officiating in Muay Thai. Muay Thai is a ring combat sport gaining in international popularity. It involves a form of boxing where competitors are encouraged to kick, punch, knee, elbow, and grapple with their opponent using full-contact strikes in an attempt to stop their opponent or gain a points victory (Myers, 2000). In a similar manner to regular boxing, a referee controls action in the ring and judges score the bout from outside of the ring. Scoring is loosely comparable to professional boxing with judges adopting a 10-point must system.

The 10-point must system involves three ringside judges each awarding 10 points to the winner of a round and fewer points to the loser. Although knockdowns and referee interventions can widen the margin, tradition limits the range of points awarded to each competitor per round. This generally involves only a single point difference between the winner and the loser of a round (10 to the winner and 9 point to the loser). Given that judges commonly award equal points to both competitors in close rounds, particularly early on in the contest, it is not uncommon for the eventual victor to win a Muay Thai fight by only a single point on a judge’s scorecard. Given overall victory is determined by a majority decision, points across judges are not totaled (e.g., if two out of three judges award a particular decision that decision stands, i.e., a win to either competitor or a draw). The judging system used has been found to be very consistent in terms of points awarded by judges and in outcome decisions. Myers et al. (2010) found that judges using the Thai judging system, only disagreed on the outcome in two out of forty-five matches and differed by no more than three points in any one match.

Using actual judges in real competition offers a number of advantages over and above the methodology employed in laboratory studies that involved the use of soccer referees. Using a live crowd noise and a live setting where judgments matter, avoids the criticism that laboratory findings are not generalizable. Using actual judges’ scores at ringside that decide the actual outcome of competition means we can begin to assess the practical significance of findings. In contrast, the fouls used in previous studies have an undetermined impact on the outcome of a match and provide less credible evidence of practical significance. The use of numerous bouts at multiple venues avoids the possibility there being any localized effect, either associated with a particular venue or competitor. As such, avoiding a comparable “Liverpool effect,” purported by some to explain the findings of Nevill and colleagues (e.g., Sutter and Kocher, 2004).

The interactive nature of a live crowd also offers the possibility of exploring social conformity effects more fully, something that is reduced considerably with the use of recorded crowd noise with no consequences to any decisions made. Conformity results from either normative or informational influence (Deutsch and Gerard, 1955). Cialdini and Goldstein (2004) proposed that individuals conform due to either a desire for accuracy, for affiliation or to maintain positive self-concept. They argued that when there is a motive for accuracy, the conformer believes others have cues for successful behavior. Yet conversely, when the aspiration of the conformer is to be accepted or valued by the group, the desire for affiliation is associated with normative conformity. Arguably both forms of conformity can influence judges’ decisions, however, the desire for group affiliation and acceptance can only be influential in live crowd contexts.

So, in an attempt to redress the issues of using recorded noise in laboratory settings, the present study assessed whether qualified Muay Thai judges scoring of bouts could be influenced by crowd noise and if such an influence would result in any home advantage effect. The hypothesis was that crowd noise would result in judges awarding inflated scores to contestants receiving the greatest level of (home) crowd support.

## Materials and Methods

### Participants

Seventeen qualified Muay Thai judges from England volunteered to take part in the present study, their experience ranging from newly qualified judges to those considered to be among the best in the UK with extensive experience of judging not only at national but also at major international shows. All participants gave their written informed consent to take part in the study prior to any testing commencing and institutional ethical approval was granted.

### Procedure

The study involved qualified Muay Thai judges officiating 30 Muay Thai bouts in one of two conditions: a “crowd noise” condition and a “no crowd noise” (noise-canceling headphones and white noise) condition. The judges scored each round of each bout using a 10-point must system, identical to that used in professional boxing, their summed scores over five rounds determining who they adjudged to be the winner of the bout (the higher score indicating the winner). The level of competition varied in standard from international bouts involving elite competitors to more novice level bouts. The crowd sizes varied from 500 to 3000, with proximity between judges and crowd varying from two to several meters. Crowd noise condition involved judges experiencing the natural crowd noise while situated at ringside, with judges in the no noise condition wearing noise-canceling headphones and listening to a track of white noise (leaving no perceptible crowd noise), also situated at ringside. Data used in the analysis only involved decisions where bouts progressed to a point’s decision and involved either a hometown boxer competing against an out-of-town boxer, or and alternatively, a UK boxer competing against a foreign opponent. Bouts involving no home favorite or where the bout was decided by way of a referee stoppage were removed entirely from the dataset. The judging system used by the judges was the 10-point must system described in the introduction. This involves judges awarding 10 to the winner and less to the loser (usually nine points to the loser unless there are knockdowns and counts by the referee, when eight or seven points are awarded – one concussive knockdown 10:8, two concussive knockdowns 10:7).

Four judges seated at ringside assessed each of the thirty, five round bouts for which data were collected, with two judges randomized to the “crowd noise” condition and two to the “no crowd noise” condition. For each bout, a record was made of the judge’s name, the condition in which they judged each bout, and the points they awarded each of the two boxers (one competing out of the red corner and the other out of the blue corner), together with a record of which boxer was the home competitor. This yielded a total of 120 judgments, with 59 in the “no noise” and 61 in the “noise” condition.

### Analysis

The difference between home and away scores over five rounds was modeled as a normal response variable. For example, a difference of −1 would indicate that the judge felt that an away (out-of-town) fighter beat the home fighter by a single point (essentially equating to being a single round ahead). Differences varied between −4 and 6, with a mean of 0.40 (standard deviation 2.47).

Importantly, in terms of data structure, single judges assessed multiple bouts and four judges judged each bout. Rather than being a hierarchical data structure, differences in score could be “classed” by judge and by bout, though judges were not nested within bouts and, likewise, bouts were not nested within judges. This type of data structure can be described as cross-classified (see Goldstein, 2010 for an introduction) and can be conveniently modeled using Markov Chain Monte Carlo methods (Browne, 2009) within MLwiN statistical software (Rasbash et al., 2009). Browne (2009) gives detailed instructions on how to implement this type of model. In general terms, there are a number of consequences of failure to correctly account for data structure, including underestimation of standard errors associated with regression coefficients (Rasbash et al., 2009). The cross-classified analysis employed in this study involved modeling “score in favor of the home side” on the basis of a single fixed factor, i.e., “noise condition.” Despite the cross-classified model, this term can be interpreted in much the same way as it would be in, for example, a standard analysis of variance (though use of a single-level ANOVA would be inappropriate because of the data structure, with observations clearly not independent). Random “between-bout” and “between-judge” variance terms accounted for the data structure, acknowledging that scores may cluster within bouts (which is highly likely where, for example, a particular fighter is clearly winning) or within judge (which could potentially happen if judges exhibited a consistent home bias). The practical significance of the findings of the cross-classified model was then addressed by assessing the potential for crowd noise effects to impact on the outcome of bouts.

## Results

### Cross-classified model

The results of the cross-classified model are shown in Table 1, with equation 1 showing the model equation. As can be seen, crowd noise had a statistically significant impact on judging. Using points in favor of the home fighter as an outcome measure (i.e., subtracting the away score from the home score) showed that exposing judges to crowd noise resulted in a difference of 0.53 points in favor of the home fighter in comparison to the no noise condition. Testing the significance of the “noise” term in the model; $\chi_{1}^{2}$ = 5.17, *p* = 0.023. As might be expected, a significant between-bout variance term indicated that scores tended to cluster by bout, whilst a non-significant between-judge term indicated that there was no evidence of scores clustering by judge. With regard to model fit, a constant only model produced a deviance information criteria of 433.53. This reduced to 429.35 when the crowd noise term was added. The deviance information criteria (Spiegelhalter et al., 2002) is a hierarchical modeling generalization of the Akaike Information Criteria. It combines measures of model accuracy and complexity, with lower values indicating the best trade-off between the two. This indicated that the model with crowd noise provided a better fit.

(1) $$\begin{array}{l} \text{Cross}-\mathrm{classified}\;\mathrm{model equation}.\\ {\text{score to Home}}_{i}∼\;N\left(\text{XB},\Omega \right)\\ {\text{score to Home}}_{i}=\beta_{0i}{\text{vcons}}_{i}+0.533\left(0.234\right){\text{noise}}_{i}\\ \beta_{0i}=0.135\left(0.443\right)+u_{0,\text{LEV2bout}\left(i\right)}^{\left(3\right)}+u_{0,\text{LEV2judge}\left(i\right)}^{\left(2\right)}+e_{0i}\\ \left[u_{0,\text{LEV2bout}\left(i\right)}^{\left(3\right)}\right]∼N\left(0,\Omega_{u}^{\left(3\right)}\right):\Omega_{u}^{\left(3\right)}=\left[4.886\left(1.500\right)\right]\\ \left[u_{0,\text{LEV2judge}\left(i\right)}^{\left(2\right)}\right]∼N\left(0,\Omega_{u}^{\left(2\right)}\right):\Omega_{u}^{\left(2\right)}=\left[0.045\left(0.089\right)\right]\\ \left[e_{0i}\right]∼N\left(0,\Omega_{e}\right):\Omega_{e}=\left[1.636\left(0.254\right)\right]\\ \text{Deviance}\left(\text{MCMC}\right)=398.339\left(120\text{ of 120 cases in use}\right) \end{array}$$

**Table 1:** **Cross-classified analysis of score in favor of home fighter by noise condition, accounting for clustering by bout and judge**.

Parameters	Level	Estimate*	Standard error
**FIXED**			
Constant		0.14	0.44
Noise condition	No noise	0.00	–
	Noise	**0.53**	**0.23**
**RANDOM**			
Between-bout variance		**4.89**	**1.50**
Between-judge variance		0.05	0.09
Between-score variance		**1.64**	**0.25**

*Values in bold are statistically significant.

### Practical significance

Despite a statistically significant difference in scores between the noise and no noise group, it does not necessarily follow that the effect is of practical significance. In the current dataset, judges awarded the same fight to different fighters in four of the thirty bouts (13.3%) of those examined. In these bouts, judges in the noise condition awarded bouts to the home boxer, whilst judges in the no crowd noise condition awarded the bout to the away boxer. So changes in the actual outcome of bouts were shown to be are possible in different crowd conditions when fights were closely contested. For example, while a bout judged without crowd noise may result in a draw, the same bout may otherwise result in a win for the home fighter in bouts judged with crowd noise present (assuming a 0.53 difference). Table 2 shows the scores awarded by judges in both the “noise” and “no noise” conditions. The shaded areas of the table show the 32 of the 120 scores (26.7%) where a change in noise condition (assuming a crowd noise effect of the size observed in the cross-classified model) could impact upon the result (just over half of the time given the 0.53 point difference between “noise” and “no noise” conditions). In the non-shaded areas (just under three-quarters of all decisions), a single point difference in favor of the home side could not impact upon the result and is therefore of little practical importance.

**Table 2:** **Points in favor of the home boxer awarded by judges in the “noise” and “no noise” conditions (shaded areas indicate decisions where a change in the noise condition could impact on the result of the bout)**.

Score to home	Condition		Total
	No noise	Noise	
−4.00	6	4	10
−3.00	5	4	9
−2.00	3	5	8
−1.00	12	6	18
0.00	5	5	10
1.00	13	10	23
2.00	4	15	19
3.00	6	5	11
4.00	3	4	7
5.00	2	1	3
6.00	0	2	2
Total	59	61	120

Close fights are relatively frequent occurrences in Muay Thai. In a larger dataset used in a previous judging study by Myers et al. (2010) which included 405 individual judging decisions from 135 Muay Thai fights randomly selected in the UK and Thailand, in 158 of the judging decisions, fighters were separated by a single point or less. Twenty-three of those decisions involved judges awarding even scores for both fighters. This suggests that crowd noise could have been a factor in 29.6% of the bouts as they were separated by a single point or less.

## Discussion

### Summary of findings

The results support the initial hypothesis presented in this paper, and suggests that crowd noise can result in judges awarding inflated scores to contestants receiving the greatest level of crowd support. The inflation in scores for the home boxer in the presence of crowd noise was statistically, and in some bouts, practically significant. On average, crowd support accounted an approximate one half-point advantage for the home fighter. While this would have little difference in bouts where one boxer was clearly dominant, it may have a real impact on the outcome of very close bouts. This was certainly the case in the present study. In the majority of bouts judges in both conditions awarded the bout to the same boxer. However, in four of the bouts, judges exposed to crowd noise awarded the decision to the hometown boxer, whilst their counterparts who did not experience the crowd noise, awarded the bout to the other competitor.

### Internal and external validity

The findings support the conclusions of previous studies that have investigated the impact of crowd noise using an experimental approach (Nevill et al., 2002; Balmer et al., 2007; Unkelbach and Memmert, 2010). The observed imbalance in favor of the home competitor also provides some support for the hypothesis that home advantage may at least in part be the result of crowd pressure on sports officials (Nevill et al., 1996) rather than merely influencing the performance of participants. The results presented here are also in accordance with the findings of archival studies that have demonstrated greater home advantage in more subjectively judged sports (Balmer et al., 2005). However, the strength of the present study and its findings relates to the fact that the observed crowd effect was the result of a live crowd environment combined with judgments made in an actual rather than simulated competition environment. So, the decisions awarded made an actual difference to the outcome of the competition and participants were aware of this. This is something that has been illusive in experiments involving just recorded crowd noise. This finding offers strong support for a crowd noise effect on sports officials’ judgment decisions, and allows the consideration of the practical significance of a crowd interaction given the high external validity. While data have been collected and analyzed previously on the crowd’s impact on official’s decisions from live events where no crowd was present (Pettersson-Lidbom and Priks, 2007), there was nevertheless low internal validity. So, the lack of an experimental approach meant that there was no deliberate control over variables, and as such while the findings may well be the result of a crowd effect, they could equally be the influence of extraneous variables.

### Why an impact of crowd noise

Given the pressures exerted by a live crowd, the findings appear to suggest judges may well have conformed to the views of the majority of spectators who were vocally supporting the home favorite. Judges in sport have been shown, and in other contexts, to be influenced by conformity biases (Scheer et al., 1983; Auweele et al., 2004; Boen et al., 2006, 2008) and this appears to be one viable explanation which supports the present study’s results. If group conformity was the reason for the differences observed between conditions, it is not clear if this was the result of normative rather than informational influences (c.f. Deutsch and Gerard, 1955). In a close fight, it is possible that judges may seek reassurance from the vocal majority in ambiguous judgment calls. However, equally, judges may have been swayed by the possibility of social sanctions from a passionate crowd at the time the decision was announced, or via “trial by web board” after the actual event in question. It has been shown that on a number of occasions following unpopular decisions, a judge’s reputation has been subject to intense scrutiny in debates on the Internet.

Although intuitively the results appear to be best explained by social conformity, it is possible that they may indeed be the result of other factors. Alternatively, a noise effect might be partly due to a noise heuristic in which the salient, yet potentially biased, judgment of the crowd provides a decision cue for referees. Balmer et al. (2007) used a repeated measures design to examine possible causes for the crowd noise effect. The research team used the same footage as they did in their original study (Nevill et al., 2002) but on this occasion also administered Competitive State Anxiety Inventory-2 (CASI2) questionnaires and gathered electrocardiogram data to determine anxiety levels. Their results suggested that crowd noise was associated with increased anxiety and mental effort, and the resulting favoritism toward the home team was purported to be used by referees as a coping strategy.

### Implications of the study

The implications of the present study can be extended beyond Muay Thai. The externally valid findings are indeed relevant to other sports. For example, in related sports such as Mixed Martial Arts (MMA) and professional boxing, the findings can be applied directly as they involve ringside judges scoring bouts using a similar 10-point must system. However, in the case of professional boxing the effect could be magnified further, given that bouts usually involve far more rounds (up to 12), than the five ordinarily competed over in Muay Thai bouts and used in this study. As such, crowd noise may in part explain the home advantage found in European championship boxing (Balmer et al., 2005). The findings are also likely to relate well to other sports where the outcome is determined by points awarded by judges. These include sports such as gymnastics and ice-skating, both of which have been subject to judging controversies over many years (e.g., Edwards, 2002; McNulty, 2004).

Though not necessarily directly applicable, the findings of this present study do offer some support for the results of the experimental studies that used recorded noise to examine crowd noise effects on soccer referees’ decisions. In sports such as soccer, experimental designs involving recorded noise may be the only practical option in which to carry out meaningful research. Investigating the real impact of a live crowd on the outcome of soccer games would undoubtedly be far more difficult. The practicality of using noise-canceling headphones successfully is one thing, but also the very variable contribution made by match officials is perhaps an even more difficult problem to address. While the decisions made by soccer referees may impact on the outcome of a game to differing degrees, they do not award points for play quality or directly assign points that contribute directly to an outcome.

It would be reasonable to assert that different judging systems are influenced to different degrees by crowd noise. Judging in Muay Thai has been found to be particularly consistent in Thailand and more recently in the UK (Myers et al., 2010). However, there are still different systems of judging used across the sport internationally (Myers, 2000). Recently it has been found that when judges use the Thai based judging system they are highly consistency in their outcome decisions (Myers et al., 2010). In the present study most of the judges had been trained previously in this type of judging system. However, in three of the four bouts where judges in different condition declared different winners, those bouts were actually judged by less experienced individuals who were not as familiar with the Thai judging system. As such, it is possible to speculate that there may be less impact on the outcome decisions of more experienced judges. So, the high level of consistency attained since UK judges began to use the Thai system may well have offered some protection against normative pressures. Furthermore, it may well be that a larger crowd effect would be evident in judging systems with a greater level of subjectivity.

### Limitations of the study

One of the limitations of the present study was the “no crowd noise” condition that was used. This involved the use of white noise. While the use of white noise is arguably useful in determining a crowd effect from a mere noise distraction, it is nevertheless not a sound that is naturally occurring within a judging context. Its use therefore, reduced the possibility of determining whether crowd noise is used as a cue by judges to help determine the relative quality of blows delivered by competitors – greater crowd noise equating to more effective strikes. Unkelbach and Memmert (2010) suggested that given the complex nature of subjective judging in sports, officials use crowd noise as an additional cue in their judgment decisions, in similar ways to cue learning in perception (e.g., Jacobs, 2002), memory judgments (Unkelbach, 2006), and decision-making (e.g., Evans et al., 2003). Another limitation was the judging system employed in our study. The 10-point must system applied is consistent and often results in only small differences between competitors, a greater variability in points may have resulted in greater differences between conditions. Therefore the transferability of results may only be applicable to sports such as boxing and MMA who use a similar 10-point must system. One possible moderating variable that was not controlled for was the potential differences in the self-efficacy of the officials involved in the study. “Refficacy,” the perceived efficacy of referees and sport officials to make split second decisions in the presence of hostile audience is receiving growing attention in the literature (Guillén and Feltz, 2011). It may well be that differing levels judge self-efficacy may mediate the effect of crowd noise on their decisions.

### Future research

Future investigations using noise-canceling headphones are possible in other sports, particularly where judges are stationary and sound is not a key component in the legitimate decision-making process in sports such as gymnastics or ice-skating. Investigations into these particular sports would offer an interesting insight into the influence of the crowd on esthetic decision-making. It would also be interesting to see if practical significance increases with greater volume or an increasingly partisan home crowd. Equally, while noise-canceling headphones may be less to practicable for mobile referees involved in sports such as soccer, an avenue for future research could be the review decisions made in cricket or rugby league.

Along with refining our understating of the different crowd effects across decision types and sports, it would also be useful to determine what makes judges more or less susceptible to crowd noise effects. Investigating the self-efficacy of officials and dispositional factors such as personality would be interesting. The possibility of determining individual level factors that may mediate crowd noise influences offer avenues for future research, the results of which may influence the training goals and the selection criteria of officials. Another area that has not been explored to any great extent is the differing influence of the content of crowd noise. While the effects of cheering and booing has been explored (e.g., Greer, 1983), other factors such as the influences on decisions of the verbal utterances of boxing seconds or protests by high profile players have not been investigated.

## Conclusion

The results of the present study suggest that crowd noise in an ecologically and externally valid setting has a statistical and, to an extent, a practically significant effect on the judgments of Muay Thai officials. The results arguably provide the first experimental evidence of the impact of live crowd noise on officials in sport. Overall, the combination of experimental, observational, and archival findings makes a compelling case for crowd influencing officials’ decisions.

## Conflict of Interest Statement

The authors declare that the research was conducted in the absence of any commercial or financial relationships that could be construed as a potential conflict of interest.
